# Relationship between Intelligence Quotient and Musical Ability in Children with Cochlear Implantation

**Published:** 2016-09

**Authors:** Simin Soleimanifar, Zahra Jafari, Masoud Motasaddi Zarandy, Houman Asadi, Hamid Haghani

**Affiliations:** 1 *Department of Audiology, School of Rehabilitation Sciences, Iran University of Medical Sciences, Tehran, Iran.*; 2 *Rehabilitation Research Center, School of Rehabilitation Sciences, Iran University of Medical Sciences, Tehran, Iran.*; 3 *Department of Otorhinolaryngology, School of Medicine, Tehran University of Medical Science, Tehran, Iran.*; 4 *Assistant Professor, School of Dramatic Arts and Music, Tehran University, Tehran, Iran.*; 5 *Department of Biostatistics, School of Management and Medical Information Science, Iran University of Medical Sciences, Tehran, Iran.*

**Keywords:** Cochlear Implants, Child, Intelligence, Music, Skill

## Abstract

**Introduction:**

Children with cochlear implants (CIs) may experience few opportunities for positive musical experiences, and musical perception is therefore often not sufficiently developed. This paper investigates and discusses the relationship between intelligence quotient (IQ) and musical ability in children with CIs compared with children with normal hearing.

**Materials and Methods:**

This was a comparative analytical study conducted in 48 children with unilateral CI and 48 normal-hearing children, 6–8 years of age, with ‘normal’ IQ and no formal music training. The average IQ score in the experimental and control groups were 105.41 and 106.31, respectively. No statistically significant differences were detected between Raven’s IQ scores in both groups. Data were collected by administering Raven's Colored Progressive Matrices IQ Tests and the Montreal Battery of Evaluation of Musical Abilities (MBEMA) Test, consisting of scale, contour, interval, rhythm, and memory sections.

**Results:**

Mean total MBEMA score in the experimental and control groups was 58.93 and 72.16 (out of 100), respectively. Significant differences were evident between scores of children with CIs in comparison with their normal-hearing peers (P≤0.001). A remarkable direct correlation between IQ and musical scores in both the control (r≥0.38) and experimental (r≥0.37) groups was observed.

**Conclusion:**

IQ has a noticeable effect on music processing and facilitates the perception of various musical elements. With regard to the mutual relationship between IQ and musical skills, this study illustrates the advantage of determining music perception scores and highlights the importance of appropriate musical intervention in order to enhance auditory neural plasticity, especially in children with cochlear implantation.

## Introduction

Music is a universal language, channelling various feelings and emotions that transcend cultural boundaries. Studies on the genetic basis of musical ability indicate that humans are born with a talent for processing feelings in music. Perception of the emotional aspects of music is considered a component of normal communication and social development (1–3). Researchers believe musical perception is a feature of human cognition (4) and a complex procedure that relies on neural networks. ‘Intelligence’ refers to the multiple mental actions involved in learning, comprehension, and reasoning. The extent of one’s intelligence can be determined by various tests, each of which are indicators of neural complexity and brain capacity (5). Intelligence quotient (IQ) reflects a broad myriad of major cognitive functions (6). One of the most important cognitive tasks is musical perception. Early exposure to music and the beginning of perception processing at the critical period of greatest brain plasticity helps facilitate the development of neural connections and could have an impact on one’s cognitive abilities, enhancing intelligence scores (7). Individuals with severe-to-profound hearing loss miss out on most of music’s acoustic characteristics (1,2,8). Although the use of cochlear implantation prostheses, based on the electrical stimulation of the auditory nerve, improves speech comprehension, this technique is unable to enhance the hearing of users to that of ‘normal’ levels (8–11).

Studies in music provide opportunities to challenge the current knowledge of brain plasticity and organization. Amusia, one of the most significant disorders of music processing, is a lack of accurate pitch and rhythm perception (3,12). Pitch perception is a basic component of normal musical development and verbal communication (1,2) and depends on a set of functions that involve the right auditory cortex, whereas processing of aspects of time, such as rhythm, is more extensive and involves bilateral neural networks (13,14). Peretz and Hyde believe that perceptual systems in individuals with amusia are unable to recognize subtle changes in pitch, due to low sensitivity to pitch deviation. Therefore, these individuals miss out on an important part of the musical experience (13). Within the past few years, research has focused on the emotional cues in music, eliciting an emotional response in CI listeners (1,2,15). The ability to recognize cues within the emotional content of music and speech has been shown to be poor in CI users (15,16). Evidence resulting from recent experiments suggests that CI users perform poorly in various temporal aspects of musical tasks when compared with their normal-hearing peers (9–11,16). However, researchers such as McDermott and Drennan discovered that rhythm perception in implant users is similar to that of normal-hearing individuals because CI devices process temporal envelopes of sound (17,18). Cochlear implantation involves the manipulation of frequency contents via sound segregation to limit frequency bands (9–11). Speech and music perception, especially in noisy conditions, remain significant challenges to CI users (19). A 2007 study by Gfeller highlighted the fact that CI recipients have a significantly poorer ability to perceive spectral cues such as pitch and timbre. This is why CI users develop high levels of speech perception with training, but cannot improve in musical fields (20).

Furthermore, researchers have indicated that learning music improves brain development and increases non-musical abilities in children. For instance, Moreno in 2009 and Chobert and François in 2013 reported that children who attended music training classes demonstrated better pitch discrimination and neural responses to music and speech, specifically. Most studies reveal that children who frequently listen to music or receive musical training show more robust brainstem responses to sound, have significantly more gray matter in several brain regions, and are more intelligent. Brain scanning technologies also reveal greater gray matter volume in several regions of the brain in children who play musical instruments (21–25).

With regard to the importance of cognitive demands in all children, particularly in the CI population, it is critical to gain knowledge about the relationships between intelligence and musical ability in order to advance basic understanding of music-induced neuroplasticity (26). Contrary to previous studies that have assessed the effects of learning music on intelligence, this study investigates the relationship between IQ and musical ability in children who have generally normal intelligence and who do not receive music training. Indeed, the present study focuses on the relationship between IQ and musical ability, regardless of previous musical training history, in order to investigate whether children without formal musical training but with higher IQ obtain higher musical scores. Further information concerning processing of musical components can be obtained through fundamental-application studies such as this. This study used a new measurement tool developed by Peretz and colleagues at the International Laboratory of Brain, Music and Sound Research in 2013 to evaluate music processing in a population of children for the first time (12).

## Materials and Methods

*Participants*

Forty-eight children with a CI were enrolled as the experimental group and 48 unimpaired girls and boys served as the control group after providing written informed consent. This research was a comparative analytical study and thus no intervention programs were introduced with respect to CI settings. The mean age of the participants in both groups was 7±0.83 years (range: 6–8 years). Fifty percent of participants were female and all were right-handed. This study was performed at two CI centers (AmirAlam Cochlear Implant Center and Iran Cochlear Implant Center) in Tehran. Data were obtained through parental completion of a bespoke history form. All CI participants in the experimental group used right unilateral Nucleus 24 device CIs with 22 activated electrodes and an ACE strategy. Exclusion criteria for both groups included a history of brain trauma, attention deficits, and learning difficulties. Lack of hearing deficit was an exclusion criteria for the control group only. Such exclusion criteria were reported by the participants’ parents, through direct observation by researchers, or through examination of medical records. One of the most important inclusion criteria among both groups was an absence of formal music training. This study was approved by the ethics committee of Iran University of Medical Sciences.

*Procedure*

The experimental session was divided into two tasks: Raven's Colored Progressive Matrices IQ Tests for Kids and Montreal Battery of Evaluation of Musical Abilities (MBEMA), which is a standardized measure of music perception in children (12, 27). Raven's Colored Progressive Matrices are nonverbal multiple choice tests designed for children between the ages of 5 and 11 consisting of 60 questions, most of which are presented on a colored background with a few on a black-and-white background. Participants are required to identify the missing element that completes a pattern. This portion of the study required approximately 40 min for completion (27).

The child’s version of the MBEMA consists of scale (skill of identifying an out-of-key note while the original contour was maintained), contour (skill of identifying pitch change of a note while the original key remained constant), interval (skill of identifying interval change of a note while the key and contour were consistent), rhythm (skill of identifying duration modifications of a grouping of notes while the number of notes was consistent), and memory (skill of melodies preservation and retention) tests (16). This standardized tool assesses musical abilities in children across different languages and cultures (12). Each test contained 20 unfamiliar tonal melody trials in 10 different keys, and two practice trials. Each test was divided into two arrangements: half included melodies that were the same, and half included melodies that were different. Each item consisted of a target and a comparison melody separated by a time interval of 1.5s. Following each item, subjects were asked to indicate verbally whether the two melodies were the same or different. The scale and memory tests were presented first and last, respectively. The final test that assesses incidental memory included 10 melodies that existed in the previous four tests, and 10 novel melodies. Participants were asked to respond ‘Yes’ (if they had previously heard the melody) or ‘No’ (if the melody was novel). No feedback was given during the test. The musical stimuli were played for each individual participant in a quiet, controlled room using a laptop, and a portable external speaker (at a fixed distance of 30cm and 0° azimuth) played at a comfortable listening level (12).

*Data analysis*

Given the abnormal score distribution for both groups (P<0.05), a Kolmogorov-Smirnov test was performed. Significant differences between the mean Raven’s IQ scores of the experimental and control groups were apparent. The musical abilities of the experimental (CI) and control groups were determined using a Mann–Whitney U-test. To investigate the relationship between Raven’s IQ scores and mean total MBEMA scores, a Spearman correlation was used. Statistical data were analyzed using SPSS.17 software, and P<0.05 was considered significant.

## Results

The sample contained 48 CI participants in the experimental group and 48 normal-hearing children in the control group aged 6–8 years (±0.83 years). The experimental group consisted of right unilateral users. Group performances on the MBEMA tests are presented in Table 1 (mean raw scores and standard deviation for experimental and control groups). Analysis indicated that the average total score in the experimental group was 58.93 (out of 100) (±10.07). The total scores of all normal-hearing participants were above the level of chance (scores of 10 or above from 20 items), with a mean of 72.16 (out of 100) (±11.73). None of the children in either group obtained a perfect score in either the subtest or the total score. Analyses revealed a significant difference in the mean of the total scores between both groups.

**Table 1:** Descriptive statistics of MBEMA for CI and control groups. Comparisons between each subtest of both groups are significant

**Mean Scores (and ±SD) with the MBEMA test**							
	n	Scale (/20)	Contour (/20)	Interval (/20)	Rhythm (/20)	Memory (/20)	Total (/100)
CI Children	48	10.91 (±1.6)	11.89 (±1.4)	11.64 (±1.56)	13.31 (±1.72)	12.37 (±1.46)	58.93 (±10.07)
NH Children	48	14.47 (±2.15)	14.39 (±2.39)	14.37 (±2.03)	15.35 (±2.1)	14.81 (±2.33)	72.16 (±11.73)
p-value		<0.001	<0.001	<0.001	<0.001	<0.001	<0.001

CI: Cochlear Implantation; MBEMA: Montreal Battery of Evaluation of Musical Abilities; NH: Normal Hearing; SD: Standard Deviation

Analysis showed that CI children performed significantly worse than the control group. The highest scores in the experimental group were recorded when measuring rhythm and memory (13.31 ±1.72 and 12.37 ±1.46, respectively). The lowest scores were recorded when measuring scale (10.96 ±1.6). Participants in the control group also performed most accurately when being measured for rhythm and memory (15.35 ±2.1 and 14.81 ±2.33, respectively).

The average IQ score in children with CIs and normal children were 105.41 (90–114) and 106.31 (90–121), respectively. No statistically significant differences were detected between Raven’s IQ scores in the experimental and normal-hearing groups according to the Mann–Whitney U-test (P=0.57).

A Spearman’s non-parametric statistical test was conducted between Raven’s IQ test scores and MBEMA subtests. This revealed a significant positive correlation between the experimental and normal groups, which means that higher scores on musical test batteries were related to higher IQ scores. Table 2 depicts the Spearman correlation coefficients between the Raven IQ scores and MBEMA subtests in both groups.

Neither group showed significant differences between either gender in each subtest in total scores (P=0.91 in the control group and P=0.59 in the CI group) or IQ tests (P=0.9 in the control group and P=0.99 in the CI group).

**Table 2:** The correlation coefficient of relationship between IQ and musical abilities (n=48 in each group

	**Items**	**Scale**	**Contour**	**Interval**	**Rhythm**	**Memory**	**Total**
Control Group	r	0.38	0.52	0.451	0.452	0.41	0.43
	p-value	0.008	<0.001	0.001	0.001	0.004	0.002
CI Group	r	0.37	0.57	0.62	0.613	0.6	0.615
	p-value	0.01	<0.001	<0.001	<0.001	<0.001	<0.001

CI: Cochlear Implantation; IQ: Intelligence Quotient

## Discussion

This study focused on the evaluation of musical perception among unimpaired and hearing-impaired children using CIs, and investigation of the relationship between music and IQ regardless of previous musical training. All unimpaired children responded accurately consistent with chance. Among children with CIs, all except three performed above the level of chance, and their results were significantly lower in all musical aspects than the control group. This finding is consistent with several previous studies investigating the music processes in CI users (1,2,28–30). These study data are also consistent with a previous study by Hopyan (16) in which the most and least accurate performance indicator of CI children was related to rhythm and scale, respectively (16). Normal-hearing peers also performed poorly on the scale subtest. In a study conducted by Peretz using the MBEMA test, results indicated lower scores of the scale subtest compared with other items. The author noted that the scale test was the most diagnostic test of music perception disorder (amusia), and by considering this criterion the prevalence of amusia increases (12).

The reason for superior performance among CI children in rhythm perception and their impaired ability to detect fine pitch differences was attributed to processing of temporal envelopes of sound as well as the device’s negligible frequency resolution capability (10, 11). Hopyan reported that a child’s ability to remember melodies requires the preservation of both pitch and rhythm. CI children have poorer memorization abilities compared with normal-hearing children due to the lack of one memory fundamental – pitch perception (16).

Mental abilities such as thinking, learning, and problem-solving are critical components of human cognition and intelligence (5). Intelligence can be divided into various subcategories, of which nonverbal intelligence is one of the most important, as it represents the ability to reason in a way that transcends all language barriers (31). In this study, children were selected who demonstrated a ‘normal’ nonverbal IQ, in the range of 90–109 as determined by Raven's Colored Progressive Matrices IQ Tests for Kids (27). A study commented on the consistency and reliability of Raven’s scores over time (32). In recent years, several studies on the relationship between music and intelligence and their influence on each other have been conducted. In an early study, Rauscher and colleagues identified an improved performance in participants’ spatial abilities after listening to the music of Mozart instead of remaining silent (32). Further studies also refer to this ‘Mozart Effect.’ Such effects were not limited to Mozart’s music only, and the effects extended beyond the individual’s spatial abilities, impacting general cognition as well. It should be noted that the Mozart Effect is not supported by a number of other studies. Peretz commented that this effect is controversial and is not related to cognitive processes, and is hard to reconcile with known principles in cognitive psychology. Indeed, there are mixed results in this field and many studies have failed to replicate this effect (33).

Almost all researchers agree that the relationship between one’s ability to learn music and one’s intelligence is cyclic and cooperative (34). To explain this controversy, it is beneficial to consider studies in two categories. The first category comprises the smaller percentage of studies and focuses on the effect intelligence has on learning and processing music. Chamorro-Premuzic, for example, described those individuals with higher IQ scores as able to use music in a more cognitive way. They are also able to process more complex elements of music. Such abilities are indicators of higher-level cognitive functioning, whereas those who listen to music purely for emotionally-driven reasons demonstrate lower IQ (35). Schellenberg established a direct connection between higher IQ and music processing. He reported that children with higher intelligence and cognition abilities have better performance and enhanced abilities to learn music (34).

Studies in the second category showed a considerable increase in intelligence and cognitive ability after direct music instruction. These studies described the learning of music in childhood and how such experiences lead to long-lasting increased intellectual ability (36). Despite such findings, one study pointed to the lack of a close association between music lessons and intelligence as the reason for a lack of ability. The author believed that the impact of music learning on cognition was related to the psychological mechanisms of the individual (37). According to Schellenberg’s findings, higher IQ scores were evident among participants with music training than among untrained individuals whose predictor variables such as gender, parent education, family income, and first languages were consistent. Also, he noted that improvement was more noticeable when considering nonverbal IQ compared with verbal IQ (34). In his 2006 article, Schellenberg described a clear lack of a relationship between IQ improvement via learning, emotional intelligence, or social skills (38). However, his recent findings in 2011 point to a general effect of music learning on intelligence, believing that this improvement was note specific to a certain type of intellectual ability and moreover, extended to academic achievement (34). In contrast, Degé and colleagues supported the idea that academic achievement in children who play or learn music was not only related to the effects of the music but was related to personality variables as well (39). Research suggests noticeable differences in frontal cortex brain function and structures among musicians compared with the average person. This is evidence of music-induced neuroplasticity and represents higher-order cognitive skill (26).

Overall, due to the importance of music, the brain has dedicated some neural space to its processing. Listening to music has an influence on auditory cortical representation (33).

Many studies have shown a link between musical training, intelligence, and verbal abilities in general and verbal memory in particular. There is some evidence for a large verbal working memory in musicians (40). IQ and executive function are related. The correlation between music and cognition may due to people with higher cognitive skills being more likely to make the cognitive effort required to learn music lessons. Listening and learning music requires focused attention and a sufficient amount of intelligence. In this model, the main reason for the direct relationship between music and IQ is that children and adults with better cognitive skills and higher IQ choose to learn music. This hypothesis suggets that children with a higher IQ are more likely to learn music lessons and to perform better on tests of cognitive abilities than children with a lower IQ (41).

In this study, a remarkable relationship was observed between IQ score and various musical abilities, with a higher IQ score leading to increases in musical skill points. Our study sits within the first category of studies described earlier with respect to the effect intelligence has on learning and processing music. Findings indicated that the strongest and the weakest relationship between IQ score and MBEMA subtests in the NH group are related to contour and scale, respectively. As observed, the positive relationship between intelligence and other subtests was stronger in children using CIs. Similar to the findings of the control group, there was a weak relationship demonstrated between IQ and scale subtests, with the strongest being related to interval. Explaining these findings should point to the ability of perception of pitch and subtle interval changes of melodic sound before the child’s birth, so that the effect of intelligence on these skills would be long-term. These components would improve in the process of plasticity. In contrast, sensitivity to musical key, such as discrimination of in-key and out-of-key changes (the scale skill) depend on exposure to a particular musical system. This skill matures later, among children 4–5 years of age. The ability to detect a deviant out-of-key note and a deviant in-key note are the same and at a lower level in infants, but the ability to detect a deviant out-of key note is better in those over 5 years of age. The scale perception ability has limited opportunity to rise to similar performance levels similar to contour and interval (12,26,42). The findings of Helmbold’s study indicate a relationship between intelligence and temporal function such as pitch discrimination that reflected specific neural information processing (43). Acton had an opposing view and reported that the modest correlations found between pitch discrimination and intelligence suggests two distinct processing pathways. Pitch perception was also thought to be independent of intelligence (44). 

As has been pointed out by Merrett, pitch perception with early training had a remarkable influence on brain structures and was an indicator of music-induced neuroplasticity progress (26).Some research has been conducted into the role of memory in intelligence fields. According to these studies, despite of a strong link between memory and intelligence, IQ and memory can be considered as two sides of a coin. IQ can influence memory, but this does not mean that higher IQ necessarily leads to superior memory ability. As frequently observed, individuals with normal intelligence could improve memory with training. What was described could be the possible reason of the modest relationship between IQ and memory in current research (34,38). This study showed that there was no remarkable difference between the performance of boys and girls on the MBEMA test battery. The findings of Peretz and colleagues in 2013 similarly demonstrated that gender had no important effect on global scores and music perception of MBEMA in French-speaking Canada, but among a Chinese population, girls performed significantly better than boys (12). Research in recent years reflects the lack of a gender effect on the results (12,13).

In general, the learning of music and performing of intellectual tasks stimulate some perceptual, cognitive, and emotional processes. Studies of various aspects of music processing provide deep insight into brain function, learning, perceiving and, ultimately, thinking and problem-solving (45). Due to the limited use of speech and language in children with CIs, we have seen that it is better to use a nonverbal intelligence test in order to evaluate IQ levels. Given the fact that various findings support the common origins of language and music, Raven's Colored Progressive Matrices IQ test would not be a perfect indicator of IQ, especially among the unimpaired. All children in both groups were in same age range group and none received any music education. It would be interesting to perform the MBEMA test on children who were musically trained in comparison with children with no musical training.

## Conclusion

This study investigated the processing of fundamental components of music and their effect on intelligence in two groups of Persian-speaking children; one group with CIs and the other with normal hearing. Children ranged in age between 6 and 8 years. A remarkable relationship between IQ and musical ability was observed, and this relationship was stronger among CI children. By exposing children to musical stimuli, children with CIs improved their musical skills, specifically rhythm and memory. Such findings highlight the importance of music learning on brain development and the occurrence of music-induced neuroplasticity. Music learning in childhood has a long-term effect on intelligence and cognitive function. Continuing research into music processing in children with hearing impairment and other special populations with a larger sample size provides increasing chances to study brain function. Design-appropriate musical inter- vention highlights a new concept, known as musical intelligence.
